# Rare diseases in Tanzania: a National Call for Action to address policy and urgent needs of individuals with rare diseases

**DOI:** 10.1186/s13023-022-02498-0

**Published:** 2022-09-05

**Authors:** Frida Kaywanga, Mohamed Zahir Alimohamed, Aneth Bella David, Daniel Maeda, Sharifa Mbarak, Togolani Mavura, Siana Nkya, Deus S. Ishengoma

**Affiliations:** 1 Department of Haematology and Blood Transfusion, Muhimbili University of Health and Allied Sciences, Dar es Salaam, Tanzania; 2 Tanzania Human Genetics Organization, Dar es Salaam, Tanzania; 3 Shree Hindu Mandal Hospital, Dar es Salaam, Tanzania; 4 Department of Genetics, University Medical Centre Groningen, University of Groningen, Groningen, The Netherlands; 5 Department of Molecular Biology and Biotechnology, University of Dar Es Salaam, Dar es Salaam, Tanzania; 6 Plant Protection Department, Swedish University of Agricultural Sciences, Alnarp, Sweden; 7 Ali Kimara Rare Diseases Foundation, Dar es Salaam, Tanzania; 8 Jakaya Mrisho Kikwete Foundation, Dar es Salaam, Tanzania; 9 Dar es Salaam University College of Education, University of Dar es Salaam, Dar es Salaam, Tanzania; 10 National Institute for Medical Research, 3 Baraka Obama Drive, P. O Box 9653, 11101 Dar es Salaam, Tanzania; 11 Faculty of Pharmaceutical Sciences, Monash University, Melbourne, Australia; 12 Harvard T.H Chan School of Public Health, Boston, MA USA

**Keywords:** Rare diseases, Rare disease day, Ali Kimara Rare Diseases Foundation (AKRDF), Tanzania Human Genetics Organization (THGO), Tanzania

## Abstract

A rare disease is generally defined as a condition which affects about 1 among 2000 people and currently, there are approximately 5000–8000 rare diseases (RDs) affecting over 400 million people world-wide. Although RDs may arise from different causes such as infections and environmental factors, about 80% are caused by genetic abnormalities. In Tanzania, there are no reports of the types of RDs, their incidence, distribution and numbers of individuals affected. In addition, there have been no strategies to map RDs in the country and develop a definition that fits the local context. Public awareness and understanding of RDs are very limited, and these lead to poor management and stigmatisation of patients. To address the ongoing problems, Tanzania joined other countries world-wide and global partners to commemorate the rare diseases day (RDD) for the first time in 2016 and subsequently every year. Unlike previous years where the RDD was organised by Ali Kimara Rare Diseases Foundation (AKRDF) with few partners, in 2020, a bigger event was co-hosted by Ali AKRDF and Tanzania Human Genetics Organization together with government representatives and other multiple partners. The organisers, government representatives and participants proposed a national “Call for Action” with the overall goal of improving the lives of patients**/**individuals with RDs. The call focuses and aims to address 17 strategic issues that are broadly categorised into four areas. These include generating demographic data of individuals with RDs; advocating for policies and guidelines for diagnosis, care, treatment and health financing; developing policies supporting public education, awareness and advocacy; and strengthening research, innovation and public–private partnerships. If adopted and implemented, the potential impacts of these recommendations will include improved access to adequate and high-quality health and education services, and policies and guidelines to address the current and future challenges facing individuals with RDs and their families.

**Supplementary Information:**

The online version contains supplementary material available at 10.1186/s13023-022-02498-0.

## Introduction

Globally, there are 5000–8000 rare diseases (RDs) that collectively affect over 400 million people worldwide [1, 2]. Although RDs may arise from different causes such as infections and environmental factors, about 80% are caused by genetic abnormalities [3]. The diseases generally vary in terms of symptoms and clinical presentations but most of them result in disabilities and poor quality of life, with phenotypes differing among ethnicities [4]. The complex nature of RDs is globally known and acknowledged [5] and has been recently recognized by the United Nations through its resolution adopted by the General Assembly in 2021 [6]. Thus, diagnosis and treatment of RDs is complicated, partly due to poor familiarity among healthcare providers [7, 8]. A lack of specific diagnostic tools and therapy lead to elongated suffering of RD patients.

In low and middle-income countries (LMICs) including Tanzania, there is limited information about RDs [5], suggesting that patients from these countries may possibly be missing in the global databases and reports. In these countries, little is known about RDs and there is a lack of data on the types of the diseases, their incidence, distribution and numbers of individuals affected. Specifically for Tanzania, there has been no efforts to map RDs and develop a nation-wide catalogue of the different conditions and definitions that fit the local context. As a result, policies and coordinated efforts that specifically target and promote access to health, education, social protection and other supportive services for individuals with RDs are lacking. In addition, there is limited public awareness and understanding of RDs which make them to be quite often associated with witchcraft and other traditional beliefs. These result in stigmatisation as well as hiding individuals living with RDs and denying them access to healthcare and other supportive services. With an already overstretched healthcare system, Tanzania and possibly other LMICs struggle to detect RDs and as a result, mis- and delayed diagnosis are common, leading to poor treatment of the conditions [9].

Currently, there are no established therapies for most of the RDs in Tanzania and for those with treatment, it is normally obtained from other countries, and it is generally expensive and out of reach for many patients [10–13]. In Tanzania, management and care of patients with RDs result in unbearable financial burden particularly to the families and close relatives. It has been noted that the health insurance schemes currently operating in Tanzania do not or partially support patients with RDs. As recently reported in Tanzania and other African countries [12], the government has given less attention to RDs due to limited understanding and appreciation of the burden of RDs and this has further left the responsibility of managing these diseases to the families.

In order to improve the quality of lives of children/individuals with RDs in Tanzania, increased public awareness and engagement are urgently needed together with appropriate policies, and treatment and supportive care systems. Thus, there is an urgent need to utilise different platforms and forums such as the commemoration of rare diseases day (RDD) to engage different stakeholders including the government to raise awareness about RDs. This paper documents and provides highlights of the 2020 RDD in Tanzania and presents the proposed “Call for Action” to promote and advocate for improved lives of individuals affected by RDs and their families, through increased access to adequate and high-quality health, education and social services, and the development of appropriate policies. The Call is a critical step because it was developed by a wide range of stakeholders including experts in human genetics and representatives of key government ministries in Tanzania who attended the 2020 RDD event. Development of this important document was informed by previous calls and initiatives [5] but retained a focus on the recommendations from the RDD 2020 event. This ensured the momentum generated by the RDD 2020, and previous events were the driving force for developing the call. All the key stakeholders in Tanzania are currently advancing important RDs agenda in line with this Call and other global initiatives and the UN resolution of 2021 [6] in order to generate solutions for the challenges that have been neglected for a long time.

## The 2020 rare diseases day in Tanzania

RDD commemorations have been held globally on the last day of February (28th or 29th) since 2008 to raise awareness among the general public and decision makers about RDs and their impacts on individuals’ lives [9]. Participants of the RDD events normally recommend to the governments and other stakeholders putting more emphasis on improving access to medical treatment and care for individuals with RDs and their families. In Tanzania, Ali Kimara Rare Disease Foundation (AKRDF) and other stakeholders organised the first RDD in 2016 and subsequently in 2019. On 29th February 2020, AKRDF and the Tanzania Human Genetics Organization (THGO) [14] jointly organised the third RDD commemorations which included government representatives and other multiple stakeholders for the first time in the country’s history (see details of the organisers and the event in Additional file 1: 1a and 1b). THGO brought in scientific expertise, advocacy and research experience to offer support to individuals with RDs and their families towards the broader mission of engaging the government and other stakeholders for increased support and awareness on different aspects of RDs in the country. The AKRDF represented individuals and children with RDs and their families. Representatives of the government of Tanzania came from the key ministries of health, education and social welfare. The organisers, government representatives and other participants utilised the opportunity to advocate for policies, strategies and interventions to address the challenges facing individuals and particularly children with RDs. The recommendations from the commemoration of the RDD 2020 have been summarised in actionable proposals in the “Call for Action” which is described below.

## Recommendations and a “Call for Action”

**Fig. 1:**
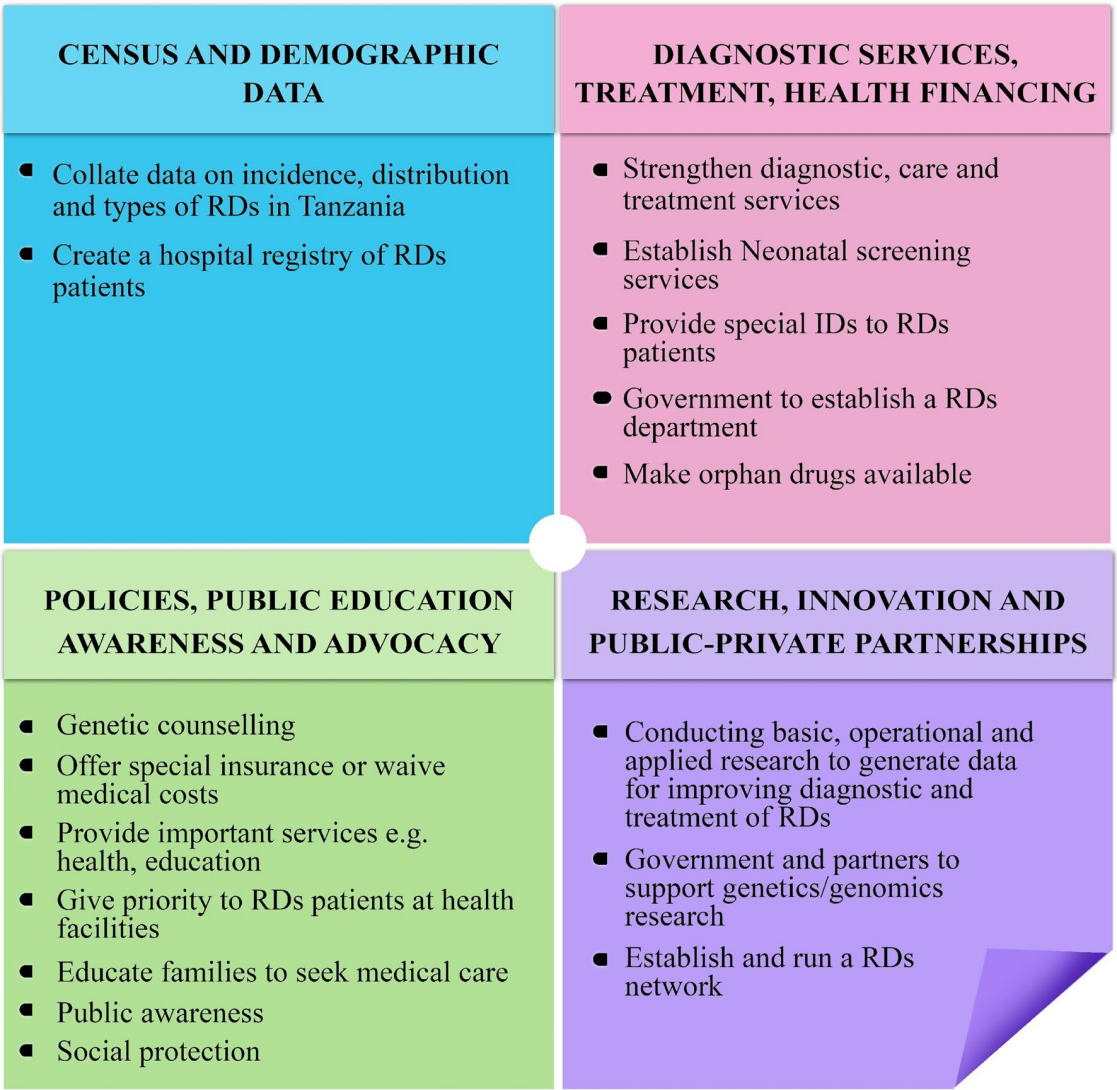
Summary of recommendations and a “Call for Action” emanating from commemoration of the RDD 2020 in Tanzania. ID, special identification of RD patients in the form of cards; RD, rare diseases; RDD, rare disease day

The “Call for Action” is focused on seventeen (17) items which are broadly grouped into four major areas. These include: (1) conducting a census survey to generate demographic data of people with RDs in the country; (2) strengthening provision of diagnostic, treatment and management services for people with RDs and improving healthcare financing; (3) developing policies that will cater for provision of public education and advocacy on RDs to subsequently reduce stigma and iv) supporting research, innovation and public private partnerships for RDs (Fig. 1). The areas covered in the Call for Action have been recommended in the call for global action for RDs in Africa [5] and recently adopted by the UN through its General Assembly resolution of 2021 [6]. Participants believe that implementation of these actionable recommendations will lay a firm foundation for infrastructure and policies/guidelines to improve management of RDs in Tanzania and potentially contribute to reducing the burden on individuals with RDs and their caretakers/families.

### Immediate census on individuals with rare diseases

The lack of data of RDs has been acknowledged and widely reported [5]. In Tanzania, there is a lack of data on the prevalence of RDs and affected individuals and this has impeded provision of social and other important services. It has also made it difficult to design policies that are inclusive enough or allocate resources for the affected groups. Hence, the government was urged to consider and support conducting an immediate census survey and baseline research across the country to obtain data that will provide an overview and the extent of the burden of different RDs in the country. The census will generate key information on the profiles of affected individuals, their geographical locations as well as diversity of diseases present. Establishing a hospital registry of children/individuals with RDs that will be linked to existing national health and education databases was also recommended. The research will obtain and collate information about immediate, short-term and long term needs of individuals and patients with RDs. The findings to be generated will provide critically needed information and recommendations that will help the government and stakeholders to potentially hasten access to healthcare, education and provision of other services to patients with RDs (Table 1).

**Table 1:** Detailed information about the “Call for Action” for RDs in Tanzania

S/N	Objective	Activities	Expected outcome	Responsible institution
1.	Rapid census and demographic data	1. To conduct an immediate census in order to obtain data that will enable the identification of children/individuals with rare diseases (RDs) in the country, including personal identity, location, type of diseases and other information	Census survey report and a database of RDs	THGO/AKRDF
		2. To establish a hospital registry for children/individuals with RDS country-wide and link it to other national databases	Registry of children/individuals with RDs	MoH/PO—RALG & THGO/AKRDF
2.	Diagnostic services, care, treatment and health financing	3. To strengthen the capacity of the health system (including health facilities) to provide diagnostic services, care and treatment to children/individuals with RDs. This should include the establishment of a formal referral system and specialized clinics with specialists for RDs	Skilled experts capable of managing patients with RDs National guidelines for referral of RD patients	MoH/THGO/Medical schools
		4. To establish neonatal screening services in maternal clinics in order to identify infants with RDs. This will ease the treatment of infants with treatable RDs and management of the condition that cannot be treated	Established services for neonatal screening	MoH/THGO/Medical schools
		5. To offer special IDs to children/individuals with RDs to enable them receive services in referral and national hospitals with less or no bureaucracy by taking into consideration that the services they require are provided thereto	IDs offered to children/individuals with RDs	MoH
		6. Establishment of a department/unit for RDs in the relevant ministries in Tanzania	Department/Unit for RDs established	MoH/MoE, Ministry of Social Welfare
		7. Orphan drugs and necessary supplies for children/individuals with RDs should be made available and accessible at all times	Orphan drugs available in specialized hospitals	MoH
3.	Policies, public education, awareness and advocacy	8. Establishment of a genetic counselling policy as well as prenatal screening of couples to enable them to plan childbearing in accordance with their genetic status	Policy and guidelines for genetic counselling and prenatal screening established	MoH
		9. The government and health Insurance operators including the National Health Insurance Fund (NHIF) should offer special insurance to children/individuals with RDs. If possible, children/individuals with RDs should get a total waiver of medical costs including costs for their medications	Policy and/or guidelines on Health Insurance of children/individuals with RDs developed and in use	MoH, NHIF, THGO/AKRDF
		10. Policies and laws should be established/reviewed in order to ensure that children/individuals with RDs are provided with their constitutional right and important services including the right to education in a conducive environment by taking into consideration of their conditions	Policy, legislation and/or guidelines on essential social services for children/individuals with RDs developed and in use	MoH, MoE, Ministry of Social Welfare, THGO/AKRDF
		11. Establishment of policies that will protect and prioritize children/people with RDs when attending health facilities, as applied to elders and pregnant women (The fee waiver policy of Tanzania)	Policy and/or guidelines on essential health services for children/individuals with RDs developed and put to practice	MoH, THGO/AKRDF
		12. To educate the general public, parents and families to bring children/individuals with RDs to the health facilities so they can access medical services and get the right treatment, counselling and other appropriate medications as well as supportive services	Guidelines for public education and provision of medical services to individuals with RDs developed and used	MoH, MoE, Ministry of Social Welfare, THGO/AKRDF/Other patients’ groups
		13. To advocate for and provide public education for the aim of eliminating stigma to children/individuals with RDs	Guidelines for public awareness about RDs developed and in use	MoH, MoE, Ministry of Social Welfare, THGO/AKRDF//Other patients’ groups
		14. Social protection experts should closely look into ways/procedures which will offer protection to children/individuals with RDs from various dangers/harms, known and unknown	Guidelines on protection of individuals with RDs developed	MoH, MoE, Ministry of Social Welfare, THGO/AKRDF
4.	Research, innovation and public private partnership	15. THGO and medical experts should provide recommendations and advice to the government and other stakeholders on what should be done in the fight against RDs in the country. This should include basic, applied and operational research on different aspects of RDs	Research on RDs undertaken Findings synthesised and disseminated Policy recommendations developed and disseminated	THGO/AKRDF/Medical schools/Research Institutions/Medical association of Tanzania
		16. The government as well as other stakeholders should promote and support the implementation of genetic/genomic research including the Tanzania Human Genome project, in order to generate important genetic data of medical and pharmacogenomic values	Genomics capacity for RDs established and strengthened Genetic/Genomics research undertaken, and findings disseminated	MoH, MoE, Ministry of Social Welfare, THGO/AKRDF/Medical schools/Research institutions
		17. To establish and run a network of stakeholders with common interest in RDs (Tanzania Rare Diseases’ Network). The network should play a key role in providing public education, awareness and advocacy in order to enhance the fight against RDs	Tanzania Rare Diseases’ Network established	MoH, MoE, Ministry of Social Welfare, THGO/AKRDF/Other patients’ groups

AKRDF, Ali Kimara Rare Diseases Foundation; MoE, Ministry of Education, MoH, Ministry of Health; NHIF, National Health Insurance Fund; PO-RALG, Ministry under The President’s Office on Regional Administrations and Local Governments Authority; THGO, Tanzania Human Genetics Organization

### Diagnostic services, care, treatment, and healthcare financing

These issues have been previously raised in other calls and reports [5]. Participants’ recommendations targeted areas such as establishment of a formal referral system and specialised clinics with specialists for RDs in order to enhance and strengthen the capacity of the healthcare system in Tanzania (including health facilities) to provide high quality diagnostic, care, treatment and preventive services to children/individuals with RDs. Establishment of neonatal screening services in maternal clinics in order to diagnose infants with known RDs was also recommended. This will allow early and timely treatment interventions where possible and commencement of appropriate management of RDs. It will also give a room for family members and caretakers to be educated on the specific conditions and their management. It was also recommended to offer special identification cards (IDs) to children/individuals with rare diseases to enable them and expedite the process of receiving healthcare services in referral and national hospitals. Additionally, establishing a department/unit for RDs within relevant ministries (such as Ministries of Health, Social Welfare and Education) in the country was recommended. The government was also advised to make orphan drugs and other essential treatment services available to RD patients.

### Policies, public education, awareness and advocacy

In this category, stakeholders recommended establishing genetic counselling centres in different parts of Tanzania which will enable patients and individuals with RDs and/or their families to get this critical service. It was also advised that services for screening of couples should be established and made available to enable them to plan childbearing with considerations of the impact of their genetic status. The government and health insurance providers were urged to offer special insurance packages to the children/individuals with RDs. If possible, service providers especially public health facilities were advised to offer a waiver of medical costs for essential medicines and other services. It was also recommended that individuals with RDs should get a priority in accessing health services at different levels of the healthcare system, similar to what is afforded to elderly and pregnant women. All these recommendations should be fitted into relevant policies to ensure their implementation and for sustainability purposes.

The government was also advised to consider and provide education services to individuals with RDs. Particularly, participants of the 2020 RDD recommended reviewing the education policy to accommodate the needs of individuals with RDs, most of whom cannot get access to regular education streams. Such actions may include giving opportunities for home-schooling, targeted education and assessments/testing as well special schools and/or facilities for children with RDs. In addition, programs on public education, engagement and awareness raising for RDs were recognized as important tools in promoting better services and eliminating stigma associated with RDs. Special social services such as counselling for patients and their caretakers as well as provision of safety and protection to children/individuals with RDs were recommended.

## Research, innovation, and public private partnerships

Research, innovation and partnerships between the private and public sectors were identified as important issues in the efforts to reduce the burden of RDs and these need to be strengthened. Through research, important clinical and pharmacogenomic data can be generated that will provide key insights on the mechanisms of RDs and pathophysiology which will assist health care professionals to understand the diseases in detail and subsequently improve diagnosis and management of most of the RDs. Additionally, it was recommended to establish and run a network of stakeholders with common interest in RDs in Tanzania (Tanzania Rare Diseases Network). The network will potentially be an important platform for providing public education, awareness and advocacy to gather and mobilize support for patients and individuals with RDs.

Finally, THGO which is comprised of experts in human genetics and as a major stakeholder and co-host of the 2020 RDD in Tanzania, was urged to be in the front line to advise the government and other stakeholders on technical and education aspects of RDs with a focus on research and translation of findings into public RDs policies, decisions and services.

## Discussion

This paper serves to document and present to the public the recommendations of the 2020 RDD that were developed and presented as a National Call for Action in Tanzania. To our knowledge, this is the first comprehensive policy recommendation for RDs in Tanzania. This call is critical and an important milestone for advocating and putting more emphasis on improving the livelihood of people living with RDs in Tanzania. We present the Call for Action as the first step to formally engage the government of Tanzania and other stakeholders to address the challenges and urgent needs of individuals with RDs in the country.

The recommendations used to develop the call covered and compiled the perspectives of a wide range of stakeholders who attended the 2020 RDD event. Participants who attended the event included patient advocacy groups, experienced scientists and medical experts, healthcare professionals and service providers involved in managing RDs, government representatives and other stakeholders with interest or supporting RDs in Tanzania. Being a historic commemoration of RDD, participants seized the moment and momentum generated by the RDD 2020 event to advance important agenda through their recommendations in order to seek solutions and address challenges facing RDs patients which have been neglected for a long time. By the time of the event, no public funding had been budgeted and spent on RDs and the insurance system in Tanzania did not support patients with RDs. The government of Tanzania admitted having a lack of awareness about RDs among the political leadership and the public. And that this has resulted in neglecting RDs, and individuals affected by these diseases. However, the government expressed the political will to implement all the recommendations from the event. We seized the opportunity to prepare and submit the recommendations to the government and other RDs stakeholders as a comprehensive “Call for Action” for implementation. Furthermore, the government through its representatives and all stakeholders expressed their willingness to support RDs in Tanzania. This opportunity had to be captured in the best way to obtain maximum benefits for RDs patients and impacts on their lives.

Despite these efforts, the implementation strategy and/or framework of this Call for Action has not been developed, and this will need to be urgently done. Coordination of multiple stakeholders involved in RDD and pursuing the agenda and recommendations presented in this call will be critical for attaining the anticipated goals. Since the key implementers of the recommendations have been clearly identified and the government has taken firm steps to implement them, developing a national plan will ensure that these and other recommendations are adequately addressed, and the impact of each step will be realised and appropriately monitored. In addition, planning of the implementation will utilise important national and international documents to develop the implementation plan/framework for Tanzania. Such resources will include among others, experience from other countries, the call for global action for RDs in Africa [5] and UN resolutions [6] to generate clear and detailed actionable activities for the government and other stakeholders.

In 2018, ​​Tanzania put in place an orphan drugs regulation [15] with the goal of controlling registration, importation and monitoring of the quality and safety of human and veterinary medicines designated as orphan medicines. It also highlights that “any patient suffering from diseases, conditions or disorders that occur rarely in the population shall be entitled to the same quality of healthcare as other patients and allowed to access orphan medicines”. While this is an important step, more efforts and engagement with the relevant departments are still needed to ensure the regulations are implemented and such drugs are imported and made available to RD patients.

To achieve the goals of this Call for Action, Tanzania will need to learn and gather experience from several countries which have made bigger strides regarding setting up national and regional policy frameworks. Such platforms and other initiatives have enabled the countries to develop key policies and govern/direct research and treatment of RDs as well as improving livelihoods of people with RDs. In the United States (US) for example, the National Organization for Rare Disorders (NORD) which was established in 1983, advocates for policy changes to improve the lives of Americans impacted by RDs at the federal and state levels [16]. Within a period of 3 decades, NORD championed many initiatives leading to remarkable achievements [17]. These include establishment of the ClinicalTrials.gov website, where all studies receiving funding from the US government must be listed. NORD and other patient advocacy groups also voiced a need for a central location where patients could learn about clinical trials for RDs. In addition, the US enacted a RDs Act of 2002, which led to establishment of the National Institute of Health (NIH) RDs Clinical Research Network, through which grant recipients are required to include patient advocacy groups in the planning and implementation of their research. Others included publication by patient advocacy groups of numerous resources to increase awareness among physicians and other medical professionals of RDs; establishment of an associate director for RDs in the US Food and Drug Authority’s (FDA) Centre for Drug Evaluation and Research, for which NORD and its member organizations provided advocacy; and many other achievements as described elsewhere [17]. For the European Union (EU), it has incorporated RDs in regional healthcare policy and set several legal frameworks to ensure commitment and adherence. The best example is the Regulation (EC) No. 141/2000 of the European Parliament and of the Council of 16 December 1999 on orphan medicinal products. The regulation provides the criteria for designating certain medicinal products as orphan drugs to prevent, diagnose and treat rare conditions and also it provides incentives for their research, development and marketing [18]. The EU has also taken important steps to enhance communication and community awareness and others such as research, gathering expertise at EU level, empowerment of patient organizations and ensuring sustainability of its strategies [19].

In other countries such as India, the government approved the National Rare Disease Policy in 2021 [20]. The policy is set to guide indigenous research and local production of medicines, lowering treatment cost of RDs and promote screening and detection of RDs at early stages. In the UK, the government and devolved administrations published the UK RDs Framework in January 2021, setting out a shared vision for addressing health inequalities and improving the lives of people living with RDs across the UK. The framework outlined four key national priorities which focus on helping patients get a final diagnosis faster; increasing awareness among healthcare professionals; better coordination of care; and improving access to specialists’ care, treatment and drugs.

Following the commemoration of the 2020 RDD the government of Tanzania has taken various steps to implement some of the recommendations presented. Such actions include, making budgetary provisions in the 2021/2022 budget of the Ministry of Health to cover different aspects of RDs. This has made financial resources available to address health challenges facing patients and individuals with RDs. Different stakeholders are making a follow-up to establish the actual areas covered in the budget and the extent of implementation at the end of the financial year in June 2022. The government has also increased access to education by providing home schooling options as well as plans to construct schools for RDs patients. Furthermore, the government has increased access to drugs and medications needed by patients with RDs as part of the national insurance schemes. Although these milestones have been achieved in a very short time, there are many challenges which are yet to be resolved to ensure high quality services to children and other individuals with RDs. This suggests that more efforts are still needed, and a stringent strategy will be required to make a strong implementation plan/framework and closely monitor the progress of implementing the recommendations proposed in this Call for Action.

## Conclusion

Although the RDD has been commemorated in Tanzania since 2016, the 2020 event was unique because it brought together government representatives and a larger number of participants and diverse stakeholders. Based on the recommendations which were made during the event, we developed actionable proposals in the form of a Call for Action to be implemented by the Government and other stakeholders. These will endeavour to formulate policies, regulations and strategies for improving the livelihood of individuals affected with RDs and their families through access to quality social services, healthcare and education. The goal of this Call for Action is to shape national policies and legal frameworks such that implementation of the recommendations will eventually lead to improved quality of life of patients and individuals living with RDs.

## Supplementary Information

**Additional file 1:** The 2020 rare diseases day in Tanzania.

## Data Availability

Not applicable.
