# Supplementary material for: Rare diseases in Tanzania: a National Call for Action to address policy and urgent needs of individuals with rare diseases
Source: Orphanet J Rare Dis. 2022 Sep 5;17:343. doi: 10.1186/s13023-022-02498-0 (PMC9446714; doi:10.1186/s13023-022-02498-0)
Supplement: Supplementary file 1 — Additional file 1: The 2020 rare diseases day in Tanzania. [file 13023_2022_2498_MOESM1_ESM.docx]

**SUPPLEMENTAL INFORMATION:**

**Rare diseases in Tanzania: A national Call for action to address policy and urgent needs of individuals with rare diseases**

Frida Kaywanga^1,2^, Mohamed Zahir Alimohamed^1,2,3,4^, Aneth David Mwakilili^2,5,6^, Daniel Maeda^2,5^, Sharifa Mbarak^7^, Togolani Mavura^7,8^, Siana Nkya^1,2,9^, Deus S. Ishengoma^2,10, 11, 12*^

^1^Department of Haematology and Blood Transfusion, Muhimbili University of Health and Allied Sciences, Dar-es-Salaam, Tanzania.

^2^Tanzania Human Genetics Organization (THGO), Dar es Salaam, Tanzania

^3^Shree Hindu Mandal Hospital, Dar es Salaam, Tanzania

^4^University of Groningen, University Medical Centre Groningen, Department of Genetics, Groningen, the Netherlands

^5^Department of Molecular Biology and Biotechnology, University of Dar es Salaam, Dar es Salaam, Tanzania

^6^Swedish University of Agricultural Sciences, Plant Protection Department, Alnarp, Sweden

^7^Ali Kimara Rare Diseases Foundation (AKRDF), Dar es Salaam, Tanzania

^8^Jakaya Mrisho Kikwete Foundation (JMKF), Dar es Salaam, Tanzania

^9^Dar es Salaam University College of Education, University of Dar es Salaam, Dar es Salaam, Tanzania

^10^National Institute for Medical Research, Dar es Salaam, Tanzania

^11^Faculty of Pharmaceutical Sciences, Monash University, Melbourne, Australia

^12^Harvard T.H Chan School of Public Health, Boston, MA, USA

*Corresponding author: Deus S. Ishengoma, 3 Baraka Obama Drive, P. O Box 9653, 11101 Dar es Salaam, Tanzania; email: [deusishe@yahoo.com](mailto:deusishe@yahoo.com)

**SUPPLEMENTAL INFORMATION 1a: ORGANIZATION FRAMEWORK AND KEY STAKEHOLDERS OF THE RDD IN TANZANIA AND KEY ISSUES FROM THE 2020 COMMEMORATION**

**Introduction**

The rare disease day (RDD) was first commemorated by 18 countries worldwide in 2008 and the number swiftly increased to 100 countries in 2019. Among them, there were five African countries; Zambia, Lesotho, Namibia, Niger and Nigeria**.** The RDD has been commemorated in Tanzania since 2016 with recorded success year after year. From 2016 to 2019, the RDD was organized by the Ali Kimara Rare disease Foundation (AKRDF). In 2020, AKRDF partnered with the Tanzania Human Genetics Organization (THGO) in organizing the RDD. The Commemoration was jointly organized by these two organizations through a National organizing Committee made of members from these organizations. This was a special event because it involved representatives of key government ministries for the first time and many partners in Tanzania. Participants of this unique event proposed recommendations which were developed into a “Call for Action” which once implemented will potentially contribute and help to address the current and future challenges facing individuals with RDs and their families.

**Ali Kimara Rare Diseases Foundation**

The AKRDF is an organization which was formed by the Kimara family in the name of Ali Kimara (a 10-year-old boy) who has been living and battling with an unknown rare disease (RD) since he was 2 years old. Ali lost his younger sibling, his sister Nasreen at the age of three due to a similar RD. The painful experiences and exposure to the reality and challenges of living with a RD, were the reasons behind the establishment of the Foundation. The purpose of the Foundation is to raise awareness and advocate for the consideration and inclusion of children and all individuals living with rare diseases in health, education and other related policies in Tanzania. The AKRDF has been recently registered in Tanzania. Additional details can be obtained from: <https://www.rarediseaseday.org/friends/ali-kimara-rare-disease-foundation/>;

**Tanzania Human Genetics Organization (**[**http://tshg.or.tz/**](http://tshg.or.tz/)**)**

Tanzania Human Genetics Organization (THGO) is a professional organization of experts involved in human genetics. It was formerly known as Tanzania Society of Human Genetics and was established in 2019 as published earlier. It has the overall goal of pursuing and advocating for research and training, increased awareness and consultancy in human genetics and related fields. It brings together scientists and medical practitioners working on human genetics and related disciplines in Tanzania to expand research and clinical services in human genetics. It fosters awareness of human genetics and genomic research in Tanzania and promoting the development of effective interventions and public policy in the field.

**The Government of the United republic of Tanzania**

The government of the United of Tanzania (both in the mainland and Zanzibar) are supporting RDs in many different ways. Coordination and implementation of RD strategies are under the Ministries of Health, Social Welfare and Education. Their support includes advancing the RDs agenda in Tanzania through development of appropriate polices. The government is also responsible for implementation of the policies through national strategic plans and annual budgets. Currently, the Government of Tanzania is focusing on health, education and supportive services. It is also supporting appropriate and better health financing through the National Health Insurance Fund to ensure that RD patients are able to access healthcare and related services equitably.

**Other patients’ advocacy groups, and organizations**

Patients’ groups which are involved in RDD and pursuit of RDs agenda include Malcom Foundation, Lukiza Foundation, Lupus Warriors Tanzania, Myasthenia Gravis Foundation of Tanzania, Epilepsy Organization of Tanzania and several sickle cell patients’ communities. These groups together with AKRDF play a key role in advocacy and raising awareness of the public on different aspects of RDs.

Other key organizations and individuals include The Jakaya Kikwete Foundation, The National Health Insurance Fund and others. The roles of these organization have been critical for the success of RDD in the past six years and generating the interest towards RDs in Tanzania. They will be involved in the implementation of the recommendations presented in the “Call for Action”.

**KEY ISSUES FROM THE 2020 RARE DISEASE DAY**

***Organizers and stakeholders***

The 2020 RDD event attracted high-level guests from the government as well as other local and international offices and organisations. The event was organized by AKRDS and THGO in collaboration with other patients’ groups, individuals, organizations and the government of the United Republic of Tanzania. Detailed information of the RDD stakeholders in Tanzania has been given above.

***Government representatives and other high-level guests***

The guest of honour for the event was Her Excellency the former Vice President and the current President of the United Republic of Tanzania, Hon. Samia Suluhu Hassan. She was represented by the former Minister for Tourism and Natural Resources, Dr. Hamisi Kigwangalla. Other high-level government officials as well as representatives from government ministries, corporates, academia, and civil societies attended the event, further adding to its significance. Some of the dignitaries who attended included the former president of the United Republic of Tanzania, Dr. Jakaya Mrisho Kikwete; former Speaker of Tanzania and Chair of the Board of the National Health Insurance Fund (NHIF), Ms. Anne Makinda and former Deputy and later Minister of Education and Vocational training in Zanzibar, Hon. Simai Mohamed Said. Furthermore, representatives of the ministers of the ministries of Health and Education of Mainland Tanzania, country offices of the World Health Organization (WHO) and United Nations International Children's Fund (UNICEF), ambassadors of UK, USA and Kuwait also attended or were represented.

***Patients’ groups***

Through AKRDF, the voice of patients and their caretakers from different patients’ groups was represented, highlighting the healthcare and social challenges they face on a daily basis. These challenges include a lack of awareness in the community about RDs that often results in stigmatization of the patients and their families and even associating RDs with witchcraft. People with RDs also face limited access to vital social services such as need-based education, poor healthcare for RDs especially diagnosis and treatment as well as economic and social burden to their families. Ms. Sharifa Mohamed, the AKRDF founder, chairperson and a mother of two children with RDs, stated on behalf of the patients’ groups, ***“I stand in front of you today, representing parents and guardians, and to share our experience raising children with rare diseases. It is my hope that through this narration and experience, we can trigger changes in systems and policies so as to bring a relief to the children with rare diseases and their families''*.** A full speech by the AKRDF is available as Supplemental Information 2.

**Researchers and academia from THGO**

THGO represented the research and academic community in the country, highlighting existing capacity in Tanzania to diagnose and manage RDs as well as the future perspectives and national potentials to combat RDs. THGO provided updates of RDs in Tanzania showing that there was limited data on the types of RDs and number of individuals affected. Based on the global data and assuming that about 10% of the people around the world are affected by RDs, THGO gave an estimate of 6 million individuals in Tanzania (total population of approximately 60million) with one of the diseases. THGO also showcased the capacity in Tanzania to diagnose RDs that is currently available in different institutions such as the Muhimbili University Genetics Laboratory (MGL) established in 2015. For improved services to individuals with RDs, THGO recommended strengthening diagnostic capacity within existing institutes, and expanding care and treatment services by training healthcare providers to better serve RDs patients. The organization also recommended improved financing and social support, increased public awareness about RDs, and forming a Tanzania RDs network for advocacy. Research and innovation, and private-public partnerships were highly recommended so as to facilitate discovery of new diagnostics and treatment options, and translation of research into policy and practices that may ease the burden of RDs in Tanzania. Specific research topics recommended by THGO included studies of the genetics of RDs and potential genetic markers, phenotyping and establishing catalogues of RDs in African populations, census surveys and establishing demographics as well as mapping RDs, development of diagnostic and screening tools and treatment options for patients with RDs.

***The government’s commitment***

The Tanzanian government, through the guest of honour, acknowledged that the attention paid to RDs has been inadequate. The guest of honour also cited the lack of reliable data about individuals with RDs as one of the factors limiting government’s intervention. As a consequence, access to essential social services such as health care and education by individuals with RDs has been either restricted or entirely absent. In the speech on behalf of the guest of honour, Dr. Kigwangalla said, ***“I acknowledge that the government has not done enough in fulfilling its responsibility to children with rare diseases. Clearly this is not deliberate but because of lack of accurate data and information about the diseases. We have forgotten them in policies and government programs in education and health by mistakenly including them in the general group of children”*.**

In addition, the government assured the organizers and participants that the recommendations from the event will be taken into consideration to ensure that the livelihood of children and individuals with RDs is improved. The guest of honour placed a special emphasis on providing access to education for individuals with RDs, as that is a right of every citizen of Tanzania regardless of their condition and living environment. In his words he said, **“*We will take all the recommendations into consideration, and we will work on them all to improve policies on providing vital services to children with RDs and their families. Let’s not ignore the fact that even children with RDs have the right to timely access better health and education services and according to their conditions”.***

# SUPPLEMENTAL INFORMATION 1b: SPEECH BY SHARIFA MOHAMED, THE CHAIRPERSON OF AKRDF AND MOTHER OF TWO CHILDREN WITH RDs

*THE SPEECH OF MS. SHARIFA MBARAK, CHAIRPERSON OF THE ALI KIMARA RARE DISEASES FOUNDATION, DURING THE WORLD RARE DISEASES DAY COMMEMORATION,*

*29 FEBRUARY 2020, DAR ES SALAAM*

*Honourable Dr. Jakaya Mrisho Kikwete, retired president of the United Republic of Tanzania and our special Guest;*

*Honourable Dr. Hamis Kigwangalla, Minister of Natural Resources and Tourism and Representing Honourable Vice President,*

*Honourable Simai Mohamed, Deputy Minister of Education and Vocational Training, Zanzibar,*

*Dr. Mohamed Zahir, General Secretary of Tanzania Society for Human Genetics, Honourable Members of Parliament,*

*Retired Ministers, Invited leaders present, Invited Guests,*

*Ladies and gentlemen.*

I am delighted today because of your presence. When we first commemorated Rare Disease Day four years ago, it was just a small event with few people who participated. We are grateful and encouraged to host you all in the commemoration of the rare disease day 2020. Personally, I did not expect to share the same table with our beloved former president Dr. Jakaya Mrisho Kikwete and honourable ministers. Thank you so much for joining us today.

Four years ago, when we commemorated the rare diseases day for the first time, I had two children, Ali and Nasreen, and both had an unknown rare disease. But as I speak today, only one child is left. Nasreen became sick a month after the rare diseases day commemorations and passed away on 21^st^ April 2016.

**Guest of honour, ladies and gentlemen,**

I stand before you to represent parents of children with rare diseases in Tanzania and share our lives and experience. We hope that by telling our stories and sharing our experience, we will persuade and promote changes in policies and systems to provide better services to children with rare diseases and their families

Today Ali is 8 years old. The journey to get here has not been easy but we are at a better place than before and probably our journey is not as bad as for others. Honestly, we are lucky because we have the support of my employer, the Central Bank of Tanzania, as well as that of our extended family and a supportive community around us. Majority of other children with rare diseases and their families are not as lucky as we are. I am therefore standing here today on behalf of those children and their families, who are voiceless and do not have the kind of platform which some of my colleagues and I have today.

**Guest of honour, ladies and gentlemen,**

Ali started to show symptoms when he was 2 years old while his four months sister Nasreen was completely healthy. He suddenly got sick one night when we were in Zanzibar, coughing and crying. We took him to the hospital in the morning where the doctors diagnosed him with mild pneumonia. However, his illness continued and upon further consultation with Professor Karim Manji, we were advised to travel to Dar es Salaam for further check-up.

We instantly left for Dar es Salaam. Upon arrival, Prof. Manji was shocked to see Ali’s condition. He was observed to have very low blood oxygen levels, but I could not notice because I had no prior experience. He was quickly admitted in the intensive care unit (ICU) at Agha Khan Hospital. I hadn't prepared myself for that moment because I thought it would be a day trip. I had no back up clothes for me and the child. My mind was focused on the Eid Al Fitr holiday which everyone was eagerly waiting for.

## Honourable Guest of Honour, Invited guests, ladies and gentlemen,

Ali’s condition worsened and we had to travel to Nairobi to seek further treatment because he needed a ventilator, and none were available at the moment. Ali and I travelled to Nairobi, and we left Nasreen behind, a four-month-old baby who was born prematurely.

In Nairobi, Ali’s condition deteriorated further, he became unconscious and had to be put on life support. His condition was unstable, and this gave us mixed feelings of hope and despair. It reached a point when even doctors started to lose hope. The only courage which made doctors continue to fight for Ali’s life came from Ali himself who never stopped fighting for his own life.

Ali’s story did not end there. There are no words that can describe his ordeal. It is a story of switching between life and death. On his first visit to Nairobi, Ali stayed in the ICU for four months. He underwent critical evacuations five times and admission in ICU more than 20 times. He became paralysed and went into a semi coma and had cardiac resuscitations five times. Sometimes, I even called home telling them Ali is gone.

Until that time, we as Ali’s parents did not know what he was suffering from. Our efforts were focusing on just saving his life. Up until now, we do not know which one of the 6000 rare diseases he is suffering from.

At the time when we had found the courage to accept Ali’s condition, Nasreen who was two-year-old also fell sick in the same manner as Ali. If one is weak in faith, they may end up asking God why me or why my children. I did not let this get to my head.

## Invited guests, Ladies and Gentlemen,

Nasreen did not get a chance to fight for her life for a long time like Ali. She passed away 11 months later. While in Zanzibar, she suddenly fell sick, and her blood oxygen levels dropped badly such that she was unable to breathe. We did not get help on time and decided to bring her to Dar es Salaam. By the time we arrived, we were told that it was too late. She was in a serious condition and had gone into a coma. Availability of Oxygen in time would have made a big difference. Oxygen only would have saved her.

One day before she died, her kidney failed, and she needed dialysis. But at that time, there was no dialysis machine for children, and she had to be transported to Nairobi by Air ambulance. We prepared ourselves so that she could be air-lifted the following day. Early in the morning, I prayed to God to make her journey easier, but if her time was up, I asked God to take her and if He wanted us to keep her, He should make it easy for her to reach Nairobi. I also told Nasreen with tears “if you still want to be with us, please be strong, but if your journey back to God is due, please go”. That day I learned that even people in a coma can hear and understand what we tell them. Despite the efforts we made to save her life, 15 minutes later she departed and that was 2016. In her short life, she was admitted in the ICU six times.

## Invited guests, Ladies and Gentlemen,

Ali and other children like him face many challenges. **First**, he had to be isolated for the **first 6 years** of his life to protect him from contracting diseases. This was a great problem for him as a child. **Second**, he often has to use a breathing machine (BiPAP) especially when he sleeps because he has chronic lung collapse. In the beginning, he used oxygen all the time, but now he only uses it when needed.

**Third**, Ali does not go to school, and he has never attended a single class in his entire life. Although Ali is being home-schooled and has shown great interest, he has not been registered as a student in Tanzania because the education policy and the current system does not recognise any other form of education apart from that obtained at school. I believe it is time that education be separated from school buildings because children can be taught anywhere including at home.

Apart from the challenges I mentioned above, there are many more problems which we encounter in our daily lives. Children with rare diseases require regular physiotherapy but we lack specialized physiotherapy centres. For starters, the children cannot use the physiotherapy centres for adults because they need to be separated. Also, these centres are mostly made for disability, which is a different case from rare diseases; you may be disabled without additional health challenges. Having a rare disease is a medical condition on its own that may lead to disability if one does not receive proper care, such as physiotherapy.

In addition to having no cure for most of the conditions, people with rare diseases suffer from opportunistic infections that often lead to admission in the ICU. The current referral system should be reviewed as it is not very friendly for children with rare diseases. When children with rare diseases need immediate medical attention, they have to begin at the primary healthcare level where expertise and equipment is limited.

From this narration and my experience, it is clear that living with children suffering from rare diseases is very costly and time consuming. At the same time, parents often stop or reduce income generating activities to take care of these children while the costs of attending to these children are usually very high. The health insurance could have been a great source of relief to children with rare diseases and their families but currently, there is no scheme for children with rare diseases.

## Invited guests, ladies and gentlemen.

We recognise and appreciate all the government efforts to improve access to healthcare services to all people. We do understand the depth and weight of the challenges the governments are facing. We have no doubt with the good will of our governments and our dear presidents, Honourable Dr. John Joseph Pombe Magufuli and Honourable Ali Mohammed Shein in improving healthcare in the country, as the government’s’ efforts are visible.

It wouldn't be fair not to recognize the progress made in diagnostic capability in the country, including the establishment of the Haematology Clinical Research and the Genetics Laboratories at Muhimbili University of Health and Allied Sciences (MUHAS). The participation of MUHAS at this event is a big relief for us.

On the other hand, we have seen a revolution for children with sickle cell diseases due to the amazing work of Prof. Julie Makani. In addition, establishment of the Tanzania Society for Human Genetics (now Tanzania Human Genetics Organization) with whom we have collaborated to organize this event brings so much hope.

## Invited guests, Ladies and Gentlemen.

You will agree with me that expressing gratitude is an opportunity to make more requests. I believe more can be done since there is government commitment. I have always believed that what is rare is precious, same as diamonds and tanzanite are, why not for rare diseases?

***Today we ask for:***

We are recommending a census of children with rare diseases that will allow creation of a register with important demographic information. The data to be generated will help to establish their number, location and the type of the conditions. This will provide evidence to support development and changes of policies on important matters that affect people with rare diseases. Perhaps their number is small so that they can be given some privileges in healthcare and special status in the health insurance schemes. The most important thing is for them to be recognized.

Children with rare diseases should receive treatment in referral healthcare centres with minimal bureaucracy given that the services they need are available at that level. Giving the Children identity cards will allow them to receive services quickly without waiting in ques or getting mixed with other patients, which usually is not good for their health.

The government should establish a centre for children with rare diseases, similar to centres for cancer and sickle cell. This will support provision of health services to the children because their needs have to be provided by a wide range of specialties. We should have people whose main responsibilities are to work with children with rare diseases. This will ensure that we have a place to go to.

We request the government and the parliament to make laws and policies so that children with rare diseases can get their constitutional right to education in any environment that is suitable to them and takes their health into count

The government should develop and implement a public awareness campaign on rare diseases. This will eliminate myths and misinformation that lead to stigmatization of people with rare diseases. The current situation discourages parents of children with rare diseases to come out openly.

## Invited guests, ladies and gentlemen;

Let me conclude by expressing gratitude to the people who have made it possible for us to face these challenges. I thank both our families for being with us from the very beginning. My sister Aysha left a 4-month-old baby to come with me to Nairobi. I thank my brothers Chau and Sultan, my aunts, Ali’s uncles and all of our extended family. Special thanks to my mother who came to live with us so as to help taking care of the children. We can't thank you enough.

I am grateful to Ali’s father, who has chosen to live for our children and shares the responsibility of taking care of them when they are sick. He has always made me believe that we are in this together, very few men are like him.

I am grateful to Ali’s doctors including Professor Karim Manji, Dr. Kija and the late Dr. Badriya Gunah of the Central Bank of Tanzania with whom we began this journey. I have not forgotten our sister and Ali’s nurse Sister Mwanaisha Shomari who has been our pillar. I am grateful to Ali’s teachers who have been teaching him without attending school. I take this opportunity to also thank my employer, the Central Bank of Tanzania, without whom today’s story would have been very different. I also, thank Jubilee health insurance and the national health insurance fund (NHIF).

, I wish to thank the board of Ali Kimara Rare Disease Foundation whose main goal is to raise public awareness on rare diseases but also to influence policy changes that will result in better livelihood of people with rare diseases. People with rare diseases are grateful for the sympathy they receive but what they need more is equitable access to social service. The board has done a lot that I wouldn't have been able to achieve alone. I am grateful to the Tanzania Society of Human Genetics for agreeing to work with us to make this event a reality. I also would like to recognise and thank the sponsors of today’s event who responded in a very short notice; CRDB Bank Limited, Serena Hotel, Mo Dewji Foundation, Save the Children, Aga Khan Hospital, Dar es Salaam Fly Ambulance, i-View Solution, Hotel Solutions, and Clouds Media as well as Azam Media who have promised to amplify our voices to reach the general public

Last but not least, I would like to thank Honourable Samia Suluhu Hassan, the Vice President of the United Republic of Tanzania for supporting our efforts to this day. I am grateful to Honourable Ummy Mwalimu, the Minister for Health, who unfortunately could not be here today due to other important responsibilities out of Dar es Salaam. Special thanks to Honourable Dr. Faustine Ndugulile, Deputy Minister for Health; and we consider him to be “The deputy minister for Health and Rare Diseases. He has been with us during all previous Rare Diseases Day commemorations except for this year, because he is attending family matters out of Dar es Salaam.

If I have missed anyone in the list, please understand and take it as a human error.

**Invited guests, Ladies and Gentlemen;**

I have spoken for a long time, and you have been patient. I didn't have a choice but to use this platform today to share our story. Thank you very much for coming, for listening to me and for supporting our cause.
